# Respiratory muscle strength is not decreased in patients undergoing cardiac surgery

**DOI:** 10.1186/s13019-016-0433-z

**Published:** 2016-03-31

**Authors:** Charlotte Urell, Margareta Emtner, Hans Hedenstrom, Elisabeth Westerdahl

**Affiliations:** Department of Neuroscience: Physiotherapy, Uppsala University, BOX 593/BMC, SE-751 24 Uppsala, Sweden; Department of Medical Sciences: Respiratory, Allergy and Sleep Research, Uppsala University, Akademiska sjukhuset, Uppsala, SE-751 85 Sweden; Department of Medical Sciences: Clinical Physiology, Uppsala University, Akademiska sjukhuset, SE-751 85 Uppsala, Sweden; Faculty of Medicine and Health, Örebro University, UFC, P.O. BOX 1613, SE-701 16 Region Örebro County Örebro, Sweden

**Keywords:** Cardiac surgery, Lung function, Median sternotomy, Respiratory muscle strength

## Abstract

**Background:**

Postoperative pulmonary impairments are significant complications after cardiac surgery. Decreased respiratory muscle strength could be one reason for impaired lung function in the postoperative period. The primary aim of this study was to describe respiratory muscle strength before and two months after cardiac surgery. A secondary aim was to describe possible associations between respiratory muscle strength and lung function.

**Methods:**

In this prospective observational study 36 adult cardiac surgery patients (67 ± 10 years) were studied. Respiratory muscle strength and lung function were measured before and two months after surgery.

**Results:**

Pre- and postoperative respiratory muscle strength was in accordance with predicted values; MIP was 78 ± 24 cmH_2_O preoperatively and 73 ± 22 cmH_2_O at two months follow-up (*p* = 0.19). MEP was 122 ± 33 cmH_2_O preoperatively and 115 ± 38 cmH_2_O at two months follow-up (*p* = 0.18). Preoperative lung function was in accordance with predicted values, but was significantly decreased postoperatively. At two-months follow-up there was a moderate correlation between MIP and FEV_1_ (*r* = 0.43, *p* = 0.009).

**Conclusions:**

Respiratory muscle strength was not impaired, either before or two months after cardiac surgery. The reason for postoperative lung function alteration is not yet known. Interventions aimed at restore an optimal postoperative lung function should focus on other interventions then respiratory muscle strength training.

## Background

Postoperative pulmonary impairments such as atelectasis, pleural effusion, pulmonary oedema, bronchospasm and pneumonia are significant complications after cardiac surgery and the reported incidence for postoperative pulmonary complications varies from 5 to 90 % depending on how the complications are defined [1–4]. The causes of pulmonary impairment are multifactorial, and one potential reason is the limited ability to take deep breaths. A restrictive and shallow breathing pattern is commonly observed after cardiac surgery performed though median sternotomy, and sternal pain is reported as one risk factor during the first postoperative days [5–7]. The respiratory muscles, and particular diaphragm, play a vital role in the breathing process. During surgery the opening of the thorax might affect nerves and muscles, but it is today unknown if decreased respiratory muscle function is a possible cause of respiratory impairment.

Preoperative respiratory dysfunction has been shown to prolong postoperative mechanical ventilation after heart valve surgery and impaired respiratory muscle strength has been described as a determinant of decreased functional capacity after coronary artery bypass grafting (CABG) [8, 9].

Reduction in respiratory muscle strength, obtained using measurement of maximal inspiratory pressure and maximal expiratory pressure generated at the mouth, has been reported during the hospital stay after cardiac surgery [10–13] however, the time course or recovery after discharge is not known. In the last decade breathing exercises aimed to influence respiratory muscle strength have been introduced in treatment of patients subjected to cardiac surgery. A number of studies have reported beneficial effects of pre- and postoperative inspiratory muscle training (IMT) in terms of restored and increased inspiratory muscle strength [10, 11], increased forced vital capacity (FVC) [14], decreased incidence of pneumonia, and decreased time in hospital [15]. In addition, some studies have shown that breathing exercises in the preoperative and immediate postoperative period increase respiratory muscle strength postoperatively [11, 15], whereas others have failed to show any effect [16].

Postoperative lung function gradually recovers, but even months after surgery a reduction of 6–13 % of preoperative spirometry has been reported [17]. Several factors that may influence postoperative lung function have been suggested; for example pleural changes, atelectasis, inflammatory reactions due to the surgery [18]. Postoperative pain sometimes highlighted to explain the decreased lung function but is rejected months after surgery [17]. The relation between lung function and respiratory muscle strength after discharge following cardiac surgery is unknown.

The primary aim of this study was to describe respiratory muscle strength before and two months after cardiac surgery. A secondary aim was to describe possible associations between respiratory muscle strength and lung function. Our hypothesis was that a significant decrease in respiratory muscle strength would be found two months after cardiac surgery. Furthermore, that there would be an association between respiratory muscle strength and lung function two months after surgery.

## Methods

The study was a prospective observational study and included data from 36 patients who had participated in a randomised controlled trial designed to study the effects of deep breathing exercises after cardiac surgery [19]. The Regional ethical review board in Uppsala, Sweden, approved the study (number 2007/160) and the patients were informed about the study procedures and completed a written informed consent process before baseline measurements.

Inclusion criteria were adult patients (>18 years), able to communicate in Swedish and scheduled for cardiac surgery (valve or CABG) via median sternotomy at the Department of Cardiothoracic Surgery at Uppsala University Hospital, Uppsala, Sweden, between April 2010 and March 2011. Patients undergoing emergency operation, previous cardiac- or lung surgery, or having renal dysfunction requiring dialysis were not included. No one required more than 24 h of mechanical ventilation, had sternum instability/infection, or was reintubated.

### Settings and procedures

Two physiotherapists and one nurse recruited the patients, who were admitted to the Department of Cardiothoracic Surgery, Uppsala University Hospital, for preoperative information. Measurements of respiratory muscle strength, spirometry and peripheral oxygen saturation (SpO_2_) were performed by biomedical scientists at the Department of Clinical Physiology, Uppsala University Hospital, before surgery and two months postoperatively. Patient characteristics were collected from medical records.

All patients received general anaesthesia and were given supplemental oxygen to maintain arterial oxygen saturation above 90 % after extubation. All patients performed deep breathing exercises hourly during the first four postoperative days. The breathing exercises were performed with a positive expiratory pressure device (PEP-device) (RIUM breathing exerciser; Rium Medical AB, Åkersberga, Sweden). The exercises included 3 sets of 10 deep breaths and the instruction were to take as deep inhalations as possible, hold for a second and than breath out in the device. The exercises were performed ones per hour when awake (daytime) during the first postoperative days. The nursing staff mobilised the patients as early as possible after extubation. On the first postoperative day, patients sat out of bed and/or stood, and on the second postoperative day, patients walked in the room, or a short distance in the corridor, and on the third postoperative day patients walked a longer distance in the corridor [19]. During the hospital stay, all patients were given analgesics according to standard routines in hospital.

### Outcomes and measurements

Respiratory muscle strength was obtained through maximal inspiratory pressure (MIP) and maximal expiratory pressure (MEP) generated at the mouth. The patient was in a seated position, breathing in a flanged mouthpiece and wearing a nose-clip. MIP was measured near residual volume after maximal exhalation and MEP was measured near total lung capacity after maximal inhalation: the highest value from three technically acceptable manoeuvres was recorded. The inspiratory- and expiratory muscle tests were standardised, as described in American Thoracic Society/European Respiratory Society (ATS/ERS) “Statement on Respiratory Muscle Testing” [20], and assessed by Jaeger Respiratory drive/Muscle strength (Intramedic, Bålsta, Sweden). Non invasive measurements for measuring MIP and MEP are widely applied and accepted [11, 21, 22]. Test-retest reliability shows in healthy people (ICC >0.8) [23] and in patients with chronic obstructive pulmonary disease (*r* = 0.97) [24]. No reliability- or validity tests are found for cardiac surgery patients. Predicted values for MIP and MEP were related to age and gender according to Evans et al. [25].

Vital capacity (VC), forced expiratory volume in 1 s (FEV_1_), and inspiratory capacity (IC) were assessed by Jaeger MasterScreenPFT/Bodybox (Intramedic, Bålsta, Sweden). Spirometry was performed in a sitting position and standardised as described in ATS/ERS “Standardization of spirometry” [26, 27]. Predicted values for VC, FEV_1,_ and IC were related to age, gender, and height according to Hedenstrom et al. [28, 29]. SpO_2_ was measured by pulse-oximetry (Rad-5v; Masimo, Irvine, CA, USA) with a probe attached to the patient’s finger.

### Statistical analysis

Version 22.0 of the SPSS software package (SPSS Inc., Chicago, IL, USA) was used for the statistical analysis. Distribution of normality was tested with Kolmogorov-Smirnov test. Student’s paired *t*-test was used to compare preoperative and postoperative values for MIP, MEP, lung function and SpO_2_. Results are expressed as mean ± SD. Pearson’s product moment correlation was used for evaluating correlations between respiratory muscle strength and lung function. A *p*-value of <0.05 was considered statistically significant.

## Results

In total, 36 cardiac surgery patients undergoing CABG (*n* = 16) and valve surgery (*n* = 20) with a median sternotomy (mean age of 67 ± 10 years) were assessed (Table 1). No significant differences were present between CABG patients and valve patients regarding patient characteristics.

**Table 1:** Pre-, peri- and postoperative patient characteristics, mean ± SD or number of patients, *n* = 36

*Preoperative*	
Age (yr)	67 ± 10
Female/male	4/32
BMI (kg/m^2^)	27 ± 4
*NYHA* I-IIIA/IIIB-IV	27/7^a^
Airflow obstruction	8
Diabetes	8
Never smoked/stopped/smokers	21/15/0
*Perioperative*	
CABG/Valve surgery	16/20
ECC time (minutes)	104 ± 44
*Postoperative*	
Postoperative mechanical time (h)	4.7 ± 2,2
Postoperative days at hospital (6-9/10-14/>14)	14/9/2^b^

*BMI* Body mass index, *CABG* Coronary artery bypass grafting, *ECC* extracorporeal circulation, *NYHA* New York Heart Association

Airflow obstruction defined as FEV_1_/VC <0.7,

^a^2 missing values

^b^1 missing value

Before surgery the respiratory muscle strength was in the normal range of predicted values (MIP 78 cmH_2_O and MEP 122 cmH_2_O) (Table 2). Two months after cardiac surgery MIP and MEP were not significantly decreased compared to preoperative values (Table 2).

**Table 2:** Maximal inspiratory pressure (MIP) and maximal expiratory pressure (MEP), mean ± SD, *n* = 36

	Preoperative	Two months follow-up	*p*-values
	cmH_2_O	cmH_2_O	
	(% predicted)	(% predicted)	
MIP	78 ± 24	73 ± 22	0.19
	(87 % ± 25 %)	(81 % ± 23 %)	
MEP	122 ± 33	115 ± 38	0.18
	(108 % ± 28 %)	(101 % ± 30 %)	

Spirometric measurements were in accordance with predicted values before surgery (VC 92 %, FEV_1_ 93 %, IC 87 %), but at two months follow up VC, FEV_1_ and IC were significantly reduced with 5, 5 and 8 % respectively, as compared to preoperative values (Table 3). Oxygenation, measured as SpO_2,_ was 97 ± 1 % preoperatively and 98 ± 1 % at the two months follow-up (*p* = 0.09).

**Table 3:** Lung function values, mean ± SD, *n* = 36

	Preoperative	Two months follow-up	*p*-values
	litre	litre	
	(% predicted)	(% predicted)	
VC	4.1 ± 0.9	3.9 ± 0.9	0.009
	(92 % ± 15 %)	(87 % ± 16 %)	
FEV_1_	3.0 ± 0.8	2.8 ± 0.7	0.001
	(93 % ± 18 %)	(88 % ± 19 %)	
IC	2.9 ± 0.7	2.6 ± 0.6	<0.001
	(87 % ± 21 %)	(79 % ± 21 %)	

*FEV*_*1*_ Forced expiratory volume in 1 s, *IC* Inspiratory capacity, *VC* Vital capacity

At two months follow-up, positive correlations were found between MIP and VC (*r* = 0.36, *p* = 0.03), FEV_1_ (*r* = 0.43, *p* = 0.009), and IC (*r* = 0.35, *p* = 0.04). No correlations were found between MEP and VC, FEV_1_ or IC.

## Discussion

No significant impairments in respiratory muscle strength were found two months after cardiac surgery, as compared to preoperative values. This is the first study to describe respiratory muscle strength after discharge in patients undergoing cardiac surgery. Riedi et al. [13] has reported an 11 % reduction in MIP five days after surgery, and Morsch et al. [12] a 36 % reduction in MIP six days after surgery. Reduced respiratory muscle strength in the early postoperative period after cardiac surgery might be due to sternal pain that affects the possibility of performing the respiratory muscle tests properly. It is still unclear whether muscle strength is assuredly affected by surgery, or whether it is masked by pain or patients’ motivation and skills to perform the test postoperatively, which might be challenging after surgery. After median sternotomy, distortion of the chest wall configuration reduces chest wall compliance and the ability to breath. Altered respiratory movements, distortion of the chest wall configuration and the reduction of the chest wall compliance might be one explanation for the decreased lung function found two months after surgery.

Two months after cardiac surgery, MIP was positively correlated with the measured lung function variables (VC, FEV_1_, IC), which indicate that this association previously found in the acute phase between MIP and FVC [30] is still present up to two months after surgery. In healthy subjects, an association between MIP and FVC has been reported [31], which supports that respiratory muscle strength and lung function is being interrelated with each other. Based on these, it would be valuable to determine whether it is actually the deep breaths performed during IMT that really affects lung volumes positively, or whether it is improvements in respiratory muscle strength that influence the lung volumes.

The significantly reduced lung function found two months postoperatively was relatively small and in accordance with predicted values for IC, VC, FEV_1_. Such a reduction maybe not affect the daily activities for the majority of the patients, but could per se be interesting in patients with considerably impaired lung function due to pulmonary disease, previous cardiothoracic surgery or other disabilities. It had been interesting to follow the patients in a longer perspective and see if the lung function returned to preoperative values.

Before surgery the respiratory muscle strength was in accordance with predicted values. The mean MIP before surgery was 78 cmH_2_O and mean MEP was 122 cmH_2_O. Both lower and higher preoperative values for MIP (66 and 84 cmH_2_O) has been reported [8, 11, 12]. Results may be dependent on different equipment for the measurement, different performance of the tests, and use of diverse predicted values for respiratory muscle strength. In this study we choose to follow the “ERS/ATS Statement on Respiratory Muscle Testing” [20] which states the values for MIP >80 cmH_2_O is not considered a clinically relevant respiratory muscle weakness. Evans et al. [25] state that MIP >50 cmH_2_O is sufficient for normal breathing and MEP >60 cmH_2_O is necessary for producing an effective cough. Moreover, if MIP falls below 60 cmH_2_O, it may still be well above the level needed to maintain normal VC [25]. Thus, there is still uncertainty about which level of respiratory muscle strength is sufficient for counteracting postoperative pulmonary impairment.

The impact of preoperatively decreased respiratory muscle strength in patients after cardiac surgery has not been fully investigated. Rodrigues et al. [8] found an association between impaired preoperative MIP and MEP (<70 % of predicted value defined by Neder et al. [32]) and the need for prolonged invasive mechanical ventilation. In abdominal, thoracic and cardiac surgery patients, MIP and/or MEP above 75 % of predictive value has been shown to be protective against the development of postoperative pulmonary complications, defined as a temperature >37.5 °C, bronchitis, atelectasis, and pneumonia during hospitalisation [33, 34]. Conversely, Riedi et al. [13] report no associations between low preoperative respiratory muscle strength and postoperative pulmonary impairments. Therefore, further studies are needed for investigating the possible role of respiratory muscle strength for the risk of development of pulmonary impairments.

The large standard deviation in MIP (73 ± 22 cmH_2_O) and MEP (115 ± 38 cmH_2_O) indicated a wide variation in respiratory muscle strength, and this large variation is in accordance with previous reports of respiratory muscle strength measurements after cardiac surgery [10, 11, 16] and in healthy subjects [20].

One limitation of this study was the small number of patients (*n* = 36) and that seriously ill patients were not included therefore the results cannot be generalised to all cardiac surgery patients. Unfortunately only few women were included in this sample. Patients with unstable angina pectoris, before surgery, were excluded in accordance with ATS/ERS recommendations [27].

We did not found significant impairments in respiratory muscle strength two months after cardiac surgery. Even if the respiratory muscle strength was not impaired it is maybe an important outcome in some patients, since an association between inspiratory respiratory muscle strength and lung function was found. Further studies are needed to identify risk patients for decreased lung function that influence pulmonary complications or physical activity ability months after surgery.

## Conclusion

Respiratory muscle strength, measured as MIP and MEP, was in accordance with predicted values both preoperatively and two months after cardiac surgery. Two months postoperatively, there was significantly decreased VC, FEV_1_ and IC. There was also an association between decreased inspiratory muscle strength and impaired lung function. The reason for the two months significant decreased lung function alteration is not yet known.
